# Effective and safe transfer of maternal antibodies persisting two months postpartum following maternal immunization with different doses of recombinant pertussis-containing vaccines

**DOI:** 10.1016/j.vaccine.2023.11.042

**Published:** 2024-01-12

**Authors:** Kulkanya Chokephaibulkit, Thanyawee Puthanakit, Surasith Chaithongwongwatthana, Niranjan Bhat, Yuxiao Tang, Suvaporn Anugulruengkitt, Chenchit Chayachinda, Sanitra Anuwutnavin, Keswadee Lapphra, Supattra Rungmaitree, Monta Tawan, Indah Andi-Lolo, Renee Holt, Librada Fortuna, Chawanee Kerdsomboon, Vilasinee Yuwaree, Souad Mansouri, Pham Hong Thai, Bruce L. Innis

**Affiliations:** a Siriraj Institute of Clinical Research (SICRES) Faculty of Medicine Siriraj Hospital, Mahidol University, 2 Wanglang Road, Bangkok 10700, Thailand; b Department of Pediatrics, Faculty of Medicine Siriraj Hospital, Mahidol University, 2 Wanglang Road, Bangkok 10700, Thailand; c Department of Pediatrics, Faculty of Medicine and Center of Excellence in Pediatric Infectious Diseases and Vaccines, Chulalongkorn University, Rama IV Road, Bangkok 10330, Thailand; d Department of Obstetrics and Gynecology, Faculty of Medicine, Chulalongkorn University, Rama IV Road, Bangkok 10330, Thailand; e Department of Obstetrics & Gynaecology, Faculty of Medicine Siriraj Hospital, Mahidol University, 2 Wanglang Road, Bangkok 10700, Thailand; f PATH, 2201 Westlake Avenue, Suite 200, Seattle, WA 98121, USA; g BioNet-Asia Co., Ltd., 19 Soi Udomsuk 37, Sukhumvit 103 Road, Bangjak, Prakanong, Bangkok 10260, Thailand

## Abstract

**Introduction:**

Recombinant acellular pertussis (ap) vaccines containing genetically inactivated pertussis toxin (PT_gen_) and filamentous hemagglutinin (FHA) with or without tetanus (TT) and diphtheria (DT) vaccines (Td) were found safe and immunogenic in non-pregnant and pregnant women. We report here maternal antibody transfer and safety data in mothers and neonates.

**Methods:**

This is the follow up of a phase 2 trial in 2019 among 400 pregnant women who randomly received one dose of recombinant pertussis-only vaccine containing 1 µg PT_gen_ and 1 µg FHA (ap1_gen_), or Td combined with ap1_gen_ (Tdap1_gen_), or with 2 µg PT_gen_ and 5 µg FHA (Tdap2_gen_), or with 5 µg PT_gen_ and 5 µg FHA (TdaP5_gen,_ Boostagen®, BioNet, Thailand) or chemically-inactivated acellular pertussis comparator (Tdap8_chem,_ Boostrix™, GSK, Belgium), either in the second or third trimester of gestation. IgG against PT, FHA, TT and DT were assessed by ELISA, PT-neutralizing antibodies (PTNA) by Chinese Hamster Ovary cell assay and safety outcomes at delivery in mothers and at birth.

**Results:**

Anti-PT and anti-FHA geometric mean concentration (GMC) ratio between infants at birth and mothers at delivery was above 1 in all groups. PT GMC in infants at birth were ≥30 IU/mL in all groups with the highest titers in infants found in TdaP5_gen_ group at birth (118.8 [95% CI 93.9–150.4]). At 2 months, PT GMC ratio to Tdap8_chem_ (98.75% CI) was significantly higher for TdaP5_gen_ (2.6 [1.7–4.0]) and comparable for other recombinant vaccines. No difference in PTNA titers at birth was observed between all groups nor between time of vaccination. Adverse events were comparable in all vaccine groups.

**Conclusions:**

BioNet licensed (TdaP5_gen_ and Tdap2_gen_) and candidate vaccines (Tdap1_gen_ and ap1_gen_) when given to pregnant women in the second or third trimester of gestation are safe and have induced passive pertussis immunity to infants.

## Highlights

• Recombinant pertussis vaccine is safe for both mother and newborn.
• There is effective transplacental antibody transfer to infants at birth.
• No difference in antibody response is shown during 2nd or 3rd trimester of pregnancy.

## 1 Introduction

Pertussis vaccination during pregnancy for prevention of early infant mortality is recommended by the World Health Organization in case of resurgence of pertussis or in countries with high or increasing infant morbidity/mortality from pertussis [1].

Pertussis immunization in pregnancy leads to active transport of maternal immunoglobulin G (IgG) antibodies across the placenta beginning in the second trimester to protect the infant during the first months of life [2].

Pertussis vaccination during pregnancy is supported by observational and randomized-controlled studies reporting significantly elevated blood antibody levels in both the mother and newborns at birth compared with those who received placebo or no vaccination, with no indication of increased risk of adverse pregnancy complications [3], and evidence that severe disease in infants may be prevented [4], [5]. Many high-income countries and countries in Latin America have adopted policies for maternal pertussis immunization (MPI), in the second or third trimester [1], [6], [7], [8] whereas, in low- and middle-income countries (LMIC), MPI is not implemented because of unclear disease burden [9], vaccine pricing, and supply constraints.

Although an immunologic correlate of protection has not been established for pertussis vaccines, the demonstrated efficacy in the context of both primary and booster immunization of vaccines containing only inactivated pertussis toxin (PT), which is a key virulence factor for *Bordetella pertussis,* indicate that immune responses to this antigen are essential [10].

Indeed, all acellular pertussis (aP) vaccines (APV) contain a PT component and nearly all of them include filamentous haemaglutinin (FHA) as well [11]. Two-component APV (PT and FHA) have been widely used in infants and have been combined with diphtheria and tetanus toxoids (DT or Td). APV differ not only in the number and concentration of the antigen components but also with regard to the bacterial clone used in production and methods of purification and detoxification (chemical or genetic) [1].

Given the potentially detrimental effects of chemical detoxification on epitope preservation [12], genetically inactivated PT (PT_gen_) containing vaccines using recombinant DNA technology have been compared to chemically inactivated acellular pertussis vaccines [13]. Earlier studies demonstrated that the genetically inactivated pertussis vaccines had higher immunogenicity than the chemically inactivated pertussis vaccines while having similar reactogenicity [11], [14].

aP5_gen_ is a two-component recombinant acellular pertussis vaccine containing 5 µg of recombinant pertussis toxin (rPT or PT_gen_) and 5 µg of FHA, developed and manufactured by BioNet (Thailand) and licensed as monovalent (Pertagen, aP5_gen_) or combined vaccines (Td-Pertagen/Boostagen®, TdaP5_gen_) in Thailand [15] and Singapore. Safety and non-inferior and superior immunogenicity of both vaccines to a licensed comparator were shown in a phase 2/3 randomized controlled trial in adolescents [16]. The long-lasting immunity induced by the two vaccines were also confirmed in a three-year pertussis antibody persistence study [17].

Due to its higher immunogenicity, it is possible that vaccines containing a lower dose of PT_gen_ could provide comparable immunity to chemically inactivated pertussis vaccines for maternal vaccination, reducing cost and making vaccine more accessible in developing countries.

Two randomized controlled trials, one in women of childbearing age and one in pregnant women, comparing monovalent (pertussis-only ap) or combined vaccines (Tdap/TdaP) with different concentrations of PT_gen_ showed the vaccines were safe and non-inferior to Tdap8_chem_ (Boostrix™; GlaxoSmithKline Biologicals, Belgium) [18], [19]. The present report describes the follow up of pregnant women at the time of delivery and their newborns at birth and before any pediatric pertussis vaccination, determining pregnancy outcomes and transferred immunity to infants.

## 2 Methods

### 2.1 Study design

This phase 2, observer-blind, randomized, active-controlled study was conducted at two sites, Siriraj Hospital and King Chulalongkorn Memorial Hospital in Thailand (Thai Clinical Trial Registry number: TCTR20180725004). Healthy pregnant women aged 18 to 40 years old with an uncomplicated singleton pregnancy were randomized to receive a single 0.5 mL dose of one of five vaccines at 20–33 weeks gestation. These five vaccines are outlined below: ap1_gen_ contains 1 µg of PT_gen_ and 1 µg of FHA; Tdap1_gen_ contains tetanus and reduced dose diphtheria toxoids (Td) (7.5 Lf of tetanus toxoid and 2.0 Lf of diphtheria toxoid) in combination with ap1_gen_; Tdap2_gen_ contains Td (7.5 Lf of tetanus toxoid and 2.0 Lf of diphtheria toxoid) in combination with 2 µg of PT_gen_ and 5 µg of FHA; TdaP5_gen_ contains Td (7.5 Lf of tetanus toxoid and 2.0 Lf of diphtheria toxoid) in combination with 5 µg of PT_gen_ and 5 µg of FHA; and Tdap8_chem_ vaccine contains Td (5 Lf of tetanus toxoid and 2.5 Lf of diphtheria toxoid) combined with 8 µg of PT, 8 µg of FHA and 2.5 μg of Pertactin. Table 1 describes the composition and batch numbers of the vaccines used in this study, that were administered by intramuscular injection preferably to the non-dominant deltoid.

**Table 1:** Study vaccines.

**Ingredient** **(per 0.5 mL dose)**	**ap1_gen_**	**Tdap1_gen_**	**Tdap2_gen_**	**TdaP5_gen_** (Boostagen®)^1^	**Tdap8_chem_** (Boostrix™)^2^
**Active ingredient**					
Tetanus toxoid	–	7.5 Lf	7.5 Lf	7.5 Lf	5 Lf
Diphtheria toxoid	–	2.0 Lf	2.0 Lf	2.0 Lf	2.5 Lf
Pertussis toxoid	1 µg^3^	1 µg^3^	2 µg^3^	5 µg^3^	8 µg^4^
Filamentous hemagglutinin	1 µg	1 µg	5 µg	5 µg	8 µg
Pertactin	–	–	–	–	2.5 µg
**Excipient**					
Adjuvant	Al(OH)_3_: 0.3 mg Al^3+^				AlPO_4:_ 0.3 mg Al^3+^Al (OH)_3_: 0.3 mg Al_3+_
NaCl	4.38 mg				4.50 mg
Water for injection	qs to 0.5 mL				qs to 0.5 mL
**Batch number**	8005A	8006A	8004A	7007A 1A	AC37B293CI

Abbreviations: Lf, limit of flocculation; mg, milligram; mL, milliliter; qs, quantum satis; µg, microgram.

1 Boostagen® is a licensed (reference) vaccine manufactured by BioNet (Thailand).

2 Boostrix™ is a licensed (comparator) vaccine manufactured by GlaxoSmithKline (GSK).

3 Genetically inactivated pertussis toxin

4 Chemically inactivated pertussis toxin

For this phase 2 study, the inclusion criteria defined the second trimester of pregnancy as beginning at week 20 up to week 26. The third trimester of pregnancy was defined as beginning at week 27 up to week 33 gestational age. Exclusion criteria are outlined in the Supplementary Appendix.

The present report outlines the immunogenicity and safety data from delivery up to 2 months post-delivery, including pregnancy outcomes.

The study was conducted in accordance with the Declaration of Helsinki and Good Clinical Practice consistent with International Conference on Harmonisation guidelines.

### 2.2 Randomization and masking

Pregnant women were randomized in a 1:1:1:1:1 ratio with block size of five (80 participants per vaccine group) to receive one dose (0.5 mL intramuscular injection) of either ap1_gen_, Tdap1_gen_, Tdap2_gen_, TdaP5_gen_ (Boostagen®), or a comparator Tdap8_chem_ (Boostrix™), according to a computer-generated (PROC PLAN, SAS® version 9.4) randomization scheme.

The trial was carried out in an observer-blind manner for the participants for all vaccine groups until 2 months after delivery, except for the ap1_gen_ group. Pregnant women assigned to this group were unblinded at Day 28 post-vaccination and were offered one dose of commercially available tetanus toxoid (TT) vaccine after blood draw and one dose of Td vaccine soon after delivery. The study pharmacist and vaccine administrator were not masked to the vaccine assignment. All other study personnel, participants, and laboratory staff were blinded to maintain the observer-blind status.

### 2.3 Study endpoints

The immunogenicity endpoints in the pregnant women include the geometric mean concentration (GMC) of anti-pertussis toxin IgG, anti-FHA, anti-tetanus, anti-diphtheria and the geometric mean titer (GMT) of PT neutralizing antibody (PTNA) titers measured at baseline and at the time of delivery, seroconversion rate defined as percentage of pregnant women with anti-PT and anti-FHA antibody concentration ≥ 4-fold increase from baseline to delivery and the percentage of pregnant women with anti-tetanus and anti-diphtheria antibody concentrations ≥0.1 IU/mL at baseline and at delivery. In infants, the immunogenicity endpoints included GMC of anti-PT, anti-FHA, anti-tetanus, anti-diphtheria and GMT of PTNA titers measured at the time of birth (cord blood or neonatal blood ≤72 hours after birth) and at 2 months of age. The immunogenicity outcomes are further outlined in the Supplementary Appendix.

Safety endpoints in the pregnant women included the frequency of unsolicited adverse events (AEs), and serious AEs (SAEs). Additional safety outcomes and assessment of the frequency of specific complications of pregnancy and delivery are summarised in the Supplementary Appendix.

Safety endpoints in the infants included the frequency of SAEs (including congenital anomalies, neonatal blood screening abnormalities, hearing deficiency detected through neonatal screening, and any other SAEs); percentage of infants with prematurity (<37 weeks of gestation), small for gestational age (SGA) (<10th percentile for gestational age), or low birthweight (<2000 grams); and the percentage with medically significant AEs.

The safety data for pregnant women and infants are reported through the time of birth.

### 2.4 Assessments

For pregnant women, blood draws were taken immediately before vaccination, 28 days after vaccination, and on the day of delivery. Approximately 5 mL cord blood sample was collected at delivery to assess maternal antibody transfer; if such a sample was not obtained, ∼3 mL of venous blood was collected from the infant within 72 hours of birth. An additional ∼ 3 mL blood sample was obtained from infants at 2 months of age.

Immunogenicity in pregnant women and infants was assessed by enzyme-linked immunosorbent assay (ELISA) for serum IgG specific for PT, FHA, diphtheria toxin (DT), and TT using blood serum [20]. Anti-PT, FHA, DT, and TT antibody testing was conducted at BioNet Human Serology Laboratory (Thailand), a nationally accredited laboratory by BLQS, DMSC Thailand in compliance with ISO 15189:2012 & 15190:20. Concentrations of serum IgG antibody against PT_gen_ and FHA were measured using validated indirect ELISA [20]. Concentrations were expressed in IU/mL, calibrated to the WHO International Standard Pertussis Antiserum (Human) 06/140. The lower limits of quantification (LLOQ; assay cut-off) were 5 IU/mL for PT-IgG and 1 IU/mL for FHA-IgG. Antibody concentrations below the assay cut-off were given arbitrary values of half the assay cut-off. The WHO Reference Reagent Pertussis Antiserum (Human) 06/142 (NIBSC, UK) was used as the positive control. Serum TT-specific and DT-specific IgG concentrations were measured using validated commercially available ELISA kits (Serion, Germany).

Sera from a subset of 24 mother-infant pairs (30%) in each vaccine group (120 total pairs) were randomly selected and assayed for PT neutralizing serum antibody by Chinese hamster ovary (CHO) cell assay at BioNet [20]. The pertussis toxin neutralizing titer was reported as IU/mL on the basis of the relative activity of the WHO International Standard Pertussis Antiserum (Human) 06/140.

Safety assessment is presented for both pregnant women and infants. The case definitions developed by the Brighton Collaboration Global Alignment of Immunization Safety in Pregnancy working groups for the assessment of AEs in mothers and infants following maternal immunization were used [21], when applicable. A Data and Safety Monitoring Board supervised enrolment and monitored the safety of maternal participants and infants throughout the trial.

### 2.5 Statistical analysis

The study includes 400 pregnant women (80 per vaccine group). The sample size was calculated based on non-inferiority test for anti-PT IgG antibody measured at the time of vaccination and 28 days after vaccination in a pooled population of subjects (400 pregnant women and 250 non-pregnant women of childbearing age [19], [22].

For analysis of immunologic results, GMCs for anti-PT, anti-FHA, anti-TT and anti-DT antibody and GMT of PT neutralizing antibody at delivery were calculated for each vaccine group along with its two-sided 95% CI, by exponentiating the corresponding log-transformed mean and its 95% CI limit. The difference in GMC or GMT between each of the study group and the comparator group were analyzed and the GMC or GMT ratio with the two-sided 95% CI were calculated based on ANOVA with Bonferroni post-hoc analysis. An alpha level less than 0.05 was applied for assigning statistical significance. At delivery, the GMC were adjusted for baseline concentration and gestational age at delivery using analysis of covariance (ANCOVA). These results are presented as ratio of adjusted GMC between each study group and comparator group. Seroconversion rates for anti-PT and anti-FHA antibody and PTNA, and seroprotection rates for anti-DT and anti-TT antibody were computed along with the corresponding exact two-sided Clopper-Pearson 95% CI for each vaccine group. The differences in the rates between each of the study group and the comparator group were calculated along with the two-sided 95% CI obtained by the Miettinen and Nurminen method.

The ratio of GMC/GMT in cord blood or neonatal blood to that in maternal participants at the time of delivery in terms of anti-PT, anti-FHA, anti-DT, and anti-TT IgG concentrations and PTNA titers (IU/mL) was computed along its two-sided 95% CI, by exponentiating the mean of the difference in log-transformed antibodies between infants and maternal participants and its 95% CI limits. The GMC of anti-PT antibodies and GMT of PTNA at the time of birth (cord blood or neonatal blood ≤72 hours after birth) in maternal participants vaccinated during the second trimester and third trimester of pregnancy in each vaccine group were computed along its two-sided 95% CI.

For maternal participants, the number and percentage experiencing medically attended AEs or SAEs reported until infants were 2 months of age were tabulated by vaccine group, severity, and causality. The frequency and percentage of participants experiencing post-vaccination pregnancy and delivery complications were also summarized by vaccine group. The frequency and percentage of infants with prematurity (<37 weeks of gestation), SGA (<10th percentile for GA), or low birthweight (<2000 g) were summarized by vaccine group. For the percentage, an exact two-sided 95% Clopper-Pearson CI was computed.

Data management and statistical analyses were performed by the Center of Excellence for Biomedical and Public Health Informatics (Bangkok, Thailand). All statistical analyses were carried out using Statistical Analysis System (SAS®) software version 9.4.

## 3 Results

### 3.1 Participant characteristics

Between February 1, 2019 and October 10, 2019, a total of 400 pregnant women were enrolled. 398 of 400 maternal participants delivered between April 4, 2019 and February 18, 2020. Two maternal participants were excluded before delivery due to informed consent withdrawal and geographical relocation. Demographic characteristics of pregnant women at baseline were similar across all vaccine groups (Table 2). At the time of vaccination, 48.3% of pregnant women were in the second trimester of pregnancy and 51.5% in the third trimester of pregnancy.

**Table 2:** Baseline characteristics of pregnant women and infants.

Pregnant women at baseline						
	**ap1_gen_**	**Tdap1_gen_**	**Tdap2_gen_**	**TdaP5_gen_**	**Tdap8_chem_**	**Total**
N	80	80	80	80	80	400
Age, mean (SD)	30.6 (4.7)	29.7 (5.1)	29.6 (6.0)	29.1 (5.7)	29.8 (5.2)	29.8 (5.3)
Height, cm, mean (SD)	158.3 (5.6)	159.5 (5.6)	158.8 (5.3)	159.4 (5.7)	160.0 (6.0)	159.2 (5.6)
Weight, kg, mean (SD)	62.1 (10.9)	65.0 (10.7)	64.6 (12.0)	65.1 (12.5)	66.7 (12.3)	64.7 (11.7)
Gestational age						
Mean (SD)	27.3 (3.3)	26.8 (3.4)	26.1 (3.3)	27.1 (3.4)*	26.8 (3.7)	26.8 (3.4)*
Second trimester, n (%)	33 (41.3)	38 (47.5)	50 (62.5)	35 (43.8)	37 (46.3)	193 (48.3)
Third trimester, n (%)	47 (58.8)	42 (52.5)	30 (37.5)	44 (55.0)	43 (53.8)	206 (51.5)
**Infants (at birth)**						
N	79	77	79	79	79	393
Male, n (%)	40 (50.6)	36 (46.8)	41 (51.9)	41 (51.9)	41 (51.9)	199 (50.6)
Female, n (%)	39 (49.4)	41 (53.3)	38 (48.1)	38 (48.1)	38 (48.1)	194 (49.4)
Birth weight, g						
Mean (SD)	3138 (333.4)	3046 (397.4)	3071 (377.9)	3048 (450.6)	3091 (281.0)	3079 (372.1)
Min-Max	2292–3870	2140–3940	2294–4430	2020–4455	2370–3940	2020–4455
Head circumference, cm						
Mean (SD)	33.8 (1.2)	33.5 (1.3)	33.7 (1.3)	33.5 (1.4)	33.8 (1.0)	33.7 (1.2)
Gestational size, n (%)						
SGA	1 (1.3)	2 (2.6)	1 (1.3)	3 (3.8)	1 (1.3)	8 (2.0)
AGA	76 (96.2)	74 (96.1)	74 (93.7)	72 (91.1)	77 (97.5)	373 (94.9)
LGA	2 (2.5)	1 (1.3)	4 (5.1)	4 (5.1)	1 (1.3)	12 (3.1)
Apgar score, n (%)						
Mean (SD) at 1 minute	8.8 (0.6)	8.8 (0.6)	8.6 (0.8)	8.8 (0.6)	8.8 (0.6)	8.7 (0.6)
Mean (SD) at 5 minutes	9.8 (0.4)	9.8 (0.5)	9.8 (0.4)	9.8 (0.4)	9.8 (0.5)	9.9 (0.4)

Abbreviations: AGA, appropriate for gestational age; cm, centimeter; g, gram; kg, kilogram; LGA, large for gestational age; min, minimum; max, maximum; N or n, number of participants; SD, standard deviation; SGA, small for gestational age.

* One pregnant woman received the vaccine at less than 20 weeks gestational age.

**Fig. 1:**
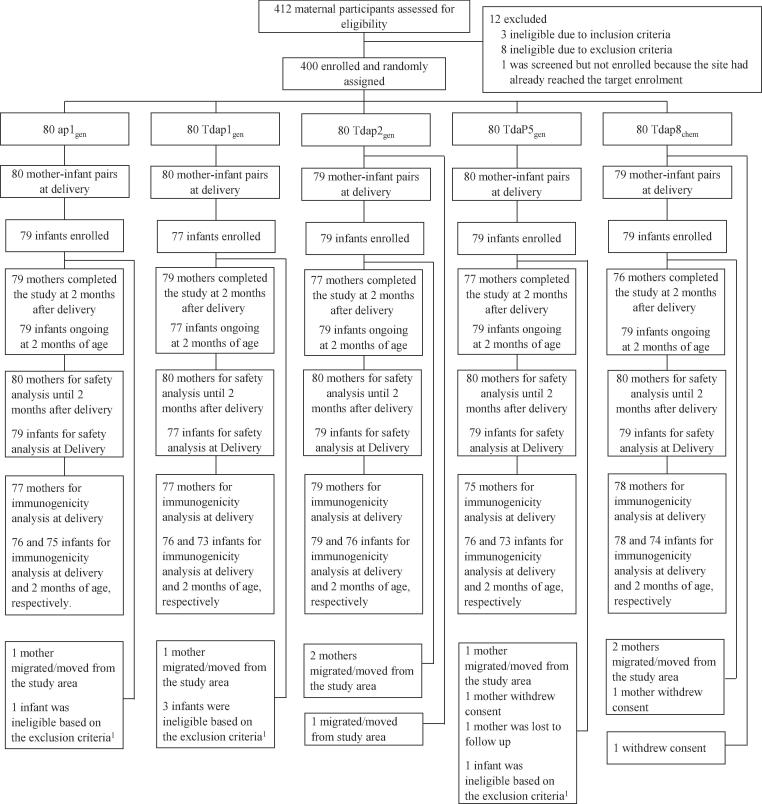
Trial profile.

In infants, demographic and other characteristics at birth were similar across all vaccine groups (Table 2). Over half of infants were male with birth weight between 2020 and 4455 grams; 94.9% of infants were assessed as appropriate for gestational age. Maternal and infant disposition at delivery and 2 months after delivery are shown in Fig. 1.

398 pregnant women were included in the safety assessment and 386 in the immunogenicity evaluation (386 participants for PT and FHA, 309 participants for DT and TT ELISA testing (for which the ap1_gen_ arm was not included), and a randomly selected subset of 115 participants for PT neutralization assay analyses) (Table 3).

**Table 3:** Safety and immunogenicity analysis populations.

**Safety and immunogenicity analysis populations**	**ap1_gen_**	**Tdap1_gen_**	**Tdap2_gen_**	**TdaP5_gen_**	**Tdap8_chem_**	**Total**
	**N (%)**	**N (%)**	**N (%)**	**N (%)**	**N (%)**	**N (%)**
**Maternal participants at delivery**						
**Safety population until delivery**	80 (100.0)	80 (100.0)	80 (100.0)	80 (100.0)	80 (100.0)	400 (100.0)
**Immunogenicity population**						
Anti-PT and anti-FHA IgG antibodies (ELISA)	77 (96.3)	77 (96.3)	79 (98.8)	75 (93.8)	78 (97.5)	386 (96.5)
Anti-TT and anti-DT IgG antibodies (ELISA)	0 (0.0)	77 (96.3)	79 (98.8)	75 (93.8)	78 (97.5)	309 (96.6)
PT-neutralizing antibody titers (CHO cell assay)	23 (95.8)	22 (91.7)	23 (95.8)	23 (95.8)	24 (100.0)	115 (95.8)
**Participants excluded from immunogenicity analysis**^1^	3 (3.8)	3 (3.8)	1 (1.3)	5 (6.3)	2 (2.5)	14 (3.5)
**Infant participants at delivery**	80	80	79	80	79	398
**Safety population at delivery**^2^	79 (98.8)	77 (96.3)	79 (100.0)	79 (98.8)	79 (100.0)	393 (98.7)
**Immunogenicity population (Cord blood or infant blood sample)**						
Anti-PT and anti-FHA IgG antibodies (ELISA)	76 (95.0)	76 (95.0)	79 (100.0)	76 (95.0)	78 (98.7)	385 (96.7)
Anti-TT and anti-DT IgG antibodies (ELISA)	0 (0.0)	76 (95.0)	79 (100.0)	76 (95.0)	78 (98.7)	313 (98.4)
PT-neutralizing antibody titers (CHO cell assay)	23 (95.8)	22 (91.7)	23 (100.0)	23 (95.8)	24 (100.0)	115 (96.6)
**Participants excluded from immunogenicity analysis**^1^**^,^**^2^	4 (5.0)	4 (5.0)	0 (0.0)	4 (5.0)	1 (1.3)	13 (3.3)
**Infant participants at 2 months of age**	79	77	79	79	79	393
**Immunogenicity population**						
Anti-PT and anti-FHA IgG antibodies (ELISA)	75 (94.9)	73 (94.8)	76 (96.2)	73 (92.4)	74 (93.7)	371 (94.4)
Anti-TT and anti-DT IgG antibodies (ELISA)	0 (0.0)	73 (94.8)	76 (96.2)	73 (92.4)	74 (93.7)	296 (94.3)
PT-neutralizing antibody titers (CHO cell assay)	23 (95.8)	21 (95.5)	23 (100.0)	24 (100.0)	24 (100.0)	115 (98.3)
**Participants excluded from immunogenicity analysis**^1^	4 (5.1)	4 (5.2)	3 (3.8)	6 (7.6)	5 (6.3)	22 (5.6)

Abbreviations: CHO, Chinese hamster ovary; DT, diphtheria toxoid; DTaP, acellular pertussis-based vaccine; DTwP, whole cell pertussis-based vaccine; ELISA, enzyme-linked immunosorbent assay; FHA, filamentous hemagglutinin; IgG, immunoglobulin G; N, number of participants; PT, pertussis toxin; Td, tetanus and diphtheria toxoid; Tdap, tetanus and diphtheria toxoid and acellular pertussis; TT, tetanus toxoid.

1 Please see Supplementary Appendix for reasons on exclusion of maternal participants from immunogenicity analysis.

2 Please see Supplementary Appendix for exclusion criteria in infants including the reasons on exclusion of infants from analysis.

Among the 398 infants born to maternal participants, 393 were included in the safety population and 385 cord blood or neonatal blood samples were included in the immunogenicity evaluation at birth. For follow up at 2 months of age, 384 eligible infants returned to the study site for safety assessment but only 371 were included in the immunogenicity evaluations.

None of the mothers nor infants were excluded for vaccine-related safety issues. Five infants were excluded from the safety population because they were ineligible as per study protocol (low birthweight or underlying medical conditions). The main reasons some pregnant women and infants were excluded from the immunogenicity evaluation are blood samples not collected, mothers receiving other vaccines before delivery and infants receiving pediatric vaccines before blood draw. Details of excluded participants are shown in Supplementary Appendix.

### 3.2 Immunogenicity of maternal and infant participants

The immunogenicity findings in mothers and in infants are summarized in Table 4. The anti-PT antibody concentrations slightly decreased from Day 28 (44.7 IU/mL – 125.9 IU/mL) to delivery (28.7 IU/mL – 92.8 IU/mL) in all groups. At delivery, the anti-PT antibody GMCs (IU/mL) in maternal participants ranged from 28.7 (95% CI 23.8–34.5) in the Tdap1_gen_ group to 92.8 (95% CI 71.4–120.5) in the TdaP5_gen_ group. The geometric mean fold rises (GMFRs) from baseline were higher in the ap1_gen_, Tdap1_gen_, Tdap2_gen,_ and TdaP5_gen_ groups than in the Tdap8_chem_ group. The adjusted anti-PT GMCs were similar in the Tdap1_gen_ and Tdap2_gen_ compared to Tdap8_chem_ group, and significantly higher in the ap1_gen_ and TdaP5_gen_ groups than in the Tdap8_chem_ group. Interestingly, the non-combined vaccine, ap1_gen_ induces higher anti-PT antibody GMC than the combined vaccine (Tdap1_gen_).

**Table 4:** Anti-PT IgG, anti-FHA IgG and PT neutralizing antibody and seroresponse rate in maternal participants at delivery, and anti-PT IgG, anti-FHA IgG and PT neutralizing antibody in infants at birth and 2 months of age.

**Maternal antibody concentrations/titers**															
	**PT**					**FHA**					**PT neutralizing antibody**				
	**ap1_gen_**	**Tdap1_gen_**	**Tdap2_gen_**	**TdaP5_gen_**	**Tdap8_chem_**	**ap1_gen_**	**Tdap1_gen_**	**Tdap2_gen_**	**TdaP5_gen_**	**Tdap8_chem_**	**ap1_gen_**	**Tdap1_gen_**	**Tdap2_gen_**	**TdaP5_gen_**	**Tdap8_chem_**
**N**	77	77	79	75	78	77	77	79	75	78	23	22	23	23	24
**Baseline** **GMC or GMT**	4.7	5.6	4.8	4.5	6.3	7.1	10.1	8.0	6.7	9.7	3.7	6.3	5.9	5.2	8.2
(IU/mL) (95% CI)	(3.7–5.9)	(4.5–6.8)	(3.8–5.9)	(3.7–5.5)	(5.0–8.0)	(5.5–9.1)	(7.8–13.0)	(6.0–10.5)	(5.2–8.7)	(7.3–12.9)	(3.2–4.3)	(4.3–9.1)	(3.8–9.0)	(4.0–6.8)	(5.3–12.7)
**Delivery** **GMC or GMT**	42.8	28.7	33.6	92.8	30.9	63.2	44.1	77.4	76.8	131.1	**28.7**	29.9	46.3	**91.2**	38.6
(IU/mL) (95% CI)	(31.9–57.3)	(23.·8–34.5)	(26.3–42.8)	(71.4–120.5)	(25.2–37.8)	(54.5–73.3)	(36.3–53.6)	(61.9–96.8)	(63.3–93.2)	(104.1–165.1)	**(14.0–59.1)**	(19.7–45.4)	(26.9–79.5)	**(57.9–143.8)**	(25.7–57.8)
**Delivery/Baseline** **GMFR**^1^	9.2	5.1	7.1	20.7	4.9	8.9	4.4	9.7	11.5	13.5	7.8	4.8	7.9	17.5	4.7
(95% CI)	(6.9–12.1)	(4.2–6.2)	(5.7–8.8)	(15.9–26.8)	(4.0–6.0)	(7.3–10.9)	(3.7–5.3)	(7.6–12.5)	(9.3–14.3)	(10.6–17.3)	(4.0–15.2)	(3.3–6.8)	(4.8–13.0)	(12.1–25.4)	(3.4–6.6)
**Adjusted GMC or GMT**	44.0	27.4	35.8	98.3	27.8	66.0	40.3	80.3	83.0	122.6	39.4	**27.8**	45.0	**97.1**	29.5
(IU/mL) (95% CI)	(35.8–54.1)	(22.3–33.6)	(29.2–44.0)	(79.7–121.2)	(22.7–34.2)	(56.3–77.4)	(34.4–47.3)	(68.6–94.0)	(70.7–97.6)	(104.7–143.6)	(25.2–61.8)	**(17.8**–**43.4)**	(29.1–69.6)	**(62.7**–**150.1)**	(19.1–45.6)
**Adjusted GMC or GMT ratio**^2^	**1**.6*	**1**.**0**	**1**.**3**	**3**.5*	**–**	**0**.5*	**0**.3*	**0**.7*	**0**.7*	**–**	**1**.**3**	**0**.**9**	**1**.**5**	**3**.3*	**–**
(98.75% or 95% CI) 3	**(1**.**1–2**.**3)**	**(0**.**7–1**.**4)**	**(0**.**9–1**.**9)**	**(2**.**4–5**.**2)**	**–**	**(0**.**4–0**.**7)**	**(0**.**3–0**.**4)**	**(0**.**5–0**.**8)**	**(0**.**5–0**.**9)**	**–**	**(0**.**7–2**.**5)**	**(0**.**5–1**.**8)**	**(0**.**8–2**.**8)**	**(1**.**8–6**.**1)**	**–**
**Infant antibody concentrations/titers**															
**Time of birth,**^4^ **N**	76	76	79	76	78	76	76	79	76	78	23	22	23	23	24
**GMC or GMT**	59.1	37.2	49.3	118.8	46.5	86.0	56.1	111.3	103.9	193.3	35.7	30.8	62.8	111.8	54.7
(IU/mL) (95% CI)	(43.4–80.5)	(30.6–45.2)	(38.6–63.0)	(93.9–150.4)	(37.9–57.0)	(73.9–100.1)	(46.5–67.7)	(89.8–137.9)	(87.4–123.6)	(154.6–241.6)	(17.0–75.0)	(20.2–47.0)	(37.7–104.6)	(64.9–192.7)	(34.4–87.1)
**Infant at birth/Mother at delivery** **GMC or GMT ratio**^5^	**1**.**4**	**1**.**3**	**1**.**5**	**1**.**3**	**1**.**5**	**1**.**4**	**1**.**3**	**1**.**4**	**1**.**4**	**1**.**5**	**1**.**2**	**1**.**0**	**1**.**4**	**1**.**2**	**1**.**4**
(95% CI)	**(1**.**3–1**.**5)**	**(1**.**2–1**.**4)**	**(1**.**4–1**.**6)**	**(1**.**2–1**.**4)**	**(1**.**4–1**.**6)**	**(1**.**3–1**.**5)**	**(1**.**2–1**.**4)**	**(1**.**3–1**.**6)**	**(1**.**2–1**.**5)**	**(1**.**4–1**.**6)**	**(0**.**9–1**.**7)**	**(0**.**8–1**.**4)**	**(1**.**2–1**.**6)**	**(1**.**0–1**.**6)**	**(1**.**2–1**.**7)**
**2 months, N**	75	73	76	73	74	75	73	76	73	74	23	21	23	24	24
**GMC or GMT**	16.5	10.5	13.9	32.8	12.5	24.3	16.6	29.8	29.7	54.8	11.8	9.0	15.9	28.7	12.5
(IU/mL) (95% CI)	(12.7–21.4)	(8.6–12.9)	(11.0–17.5)	(25.7–42.0)	(10.1–15.5)	(20.9–28.3)	(13.8–19.8)	(23.8–37.4)	(25.0–35.2)	(43.2–69.3)	(6.4–21.5)	(6.5–12.5)	(9.1–27.9)	(18.1–45.6)	(8.0–19.6)
**GMC or GMT ratio**^2^	**1.3**	**0.8**	**1.1**	2.6*	**–**	0.4*	0.3*	0.5*	0.5*	**–**	**0.9**	**0.7**	**1.3**	**2.3**	**–**
(98.75% CI)	**(0.9**–**2.0)**	**(0.6**–**1.3)**	**(0.7**–**1.7)**	**(1.7**–**4.0)**	**–**	**(0.3**–**0.6)**	**(0.2**–**0.4)**	**(0.4**–**0.8)**	**(0.4**–**0.8)**	**–**	**(0.4**–**2.2)**	**(0.3**–**1.7)**	**(0.6**–**3.0)**	**(1.0**–**5.3)**	**–**
**Maternal seroresponse rate (4-fold increase from baseline)**															
**Delivery, N**	77	77	79	75	78	77	77	79	75	78	23	22	23	23	24
**n (%)**	57 (74.0)	49 (63.6)	58 (73.4)	69 (92.0)	45 (57.7)	62 (80.5)	43 (55.8)	62 (78.5)	65 (86.7)	67 (85.9)	14 (60.9)	14 (63.6)	16 (69.6)	21 (91.3)	14 (58.3)
(95% CI)	(62.8–83.4)	(51.9–74.3)	(62.3–82.7)	(83.4–97.0)	(4.0–68.8)	(69.9–88.7)	(44.1–67.2)	(67.8–86.9)	(76.8–93.4)	(76.2–92.7)	(38.5–80.3)	(40.7–82.8)	(47.1–86.8)	(72.0–98.9)	(36.6–77.9)
**Difference**^6^**(%)**	**16**.**3**	**5**.**9**	**15**.**7**	**34**.**3**	**–**	**–5**.**4**	**–30**.**1**	**–7**.**4**	**0**.**8**	**–**	**2**.**5**	**5**.**3**	**11**.**2**	**33**.**0**	**–**
(95% CI)	**(1**.**4–30**.**7)**	**(-9**.**4–21**.**1)**	**(0**.**8**–**30**.**0)**	**(21**.**4**–**46**.**6)**	**–**	**(–17**.**5–6**.**6)**	**(-43**.**2–16**.**1)**	**(–19**·**6–4**.**7)**	**(–10**.**6–12**.**1)**	**–**	**(–25**.**1–29**.**7)**	**(–22**.**7–32**.**3)**	**(–16**.**4–37**.**2)**	**(8**.**5–54**.**8)**	**–**

Abbreviations: ANCOVA, analysis of covariance; CI, confidence interval; FHA, filamentous hemagglutinin; GA, gestational age; GMC, geometric mean concentration for PT and FHA;

GMFR, geometric mean fold rise; GMT, geometric mean titer for PT neutralizing antibody; IU, international unit; mL, milliliter; N or n, number of participants; PT, pertussis toxin.

1 Geometric mean fold rise (GMFR) is geometric mean of the ratios of antibody concentration/titer at delivery to antibody concentration/titer at baseline.

2 The ratio of study group (ap1_gen_/Tdap1_gen_/Tdap2_gen_/TdaP5_gen_) to comparator group (Tdap8_chem_).

3 PT: The ratio of adjusted GMC between each study group and comparator group with 98.75% CI was calculated based on ANCOVA model, adjusted for concentrations at baseline and GA at baseline. FHA: The ratio of adjusted GMC between each study group and comparator group with 95% CI was calculated based on ANCOVA model, adjusted for concentrations at baseline and GA at baseline. PT neutralizing: The ratio of adjusted GMT between each study group and comparator group with 95% CI was calculated based on ANCOVA model, adjusted for concentrations at baseline.

4 Time of birth (cord blood or neonatal blood within 72 hours after birth).

5 The ratio of infant antibody concentration at time of birth (cord blood or neonatal blood within 72 hours after birth) to the mother's antibody concentration at time of delivery.

6 Difference in seroresponse rate between study group and comparator group.

* The GMC, adjusted GMC, GMT or adjusted GMT between study group and comparator group is significantly different (*p* < 0.05).

Analyses of anti-FHA IgG concentrations in maternal participants are summarized in Table 4. A trend towards a slight decrease in anti-FHA antibody concentrations from Day 28 to delivery in all vaccine groups was seen. Anti-FHA IgG GMC was higher in the Tdap8_chem_ group than for the other vaccine groups.

At delivery, PTNA (IU/mL) in mothers ranged from 28.7 (95% CI 14.0–59.1) in the ap1_gen_ group to 91.2 (95% CI 57.9–143.8) in the TdaP5_gen_ group (Table 4). The GMFRs from baseline were higher in the ap1_gen_, Tdap2_gen,_ and TdaP5_gen_ groups than in the Tdap1_gen_ and Tdap8_chem_ groups.

The adjusted PT neutralizing GMT ratio of each study vaccine to the comparator vaccine (Tdap8_chem_) showed that the adjusted PT neutralizing GMT was similar in the ap1_gen_, Tdap1_gen_ Tdap2_gen_ groups compared to the Tdap8_chem_ group, and significantly higher in the TdaP5_gen_ groups than in the Tdap8_chem_ group.

At delivery, the difference in anti-PT seroresponse rates between study groups and the comparator group was highest for TdaP5_gen_ (34.3% [95 % CI 21.4–46.6]) and lowest for Tdap1_gen_ (5.9% [95% CI −9.4–21.1]) (Table 4). For anti-FHA, the differences in the seroresponse rates were highest for TdaP5_gen_ (0.8 [95% CI −10.6–12.1]) and lowest for Tdap1_gen_ (-30.1 [95% CI −43.2–16.1]). For PTNA, the difference in the rates were highest for TdaP5_gen_ (33.0 [95% CI 8.5–54.8]) and lowest for ap1_gen_ (2.5 [95% CI −25.1–29.7]).

Analyses of anti-DT and anti-TT IgG concentrations in maternal participants are summarized in Table 5. Increases in anti-DT and anti-TT IgG concentrations within the 28 days after vaccination were observed (unpublished data), with a trend towards a slight decrease in anti-DT and anti-TT IgG concentrations from Day 28 to delivery. At delivery, the anti-TT seroprotection rates were 100% for all groups, while anti-DT seroprotection rates were above 90% in all study vaccines including the comparator.

**Table 5:** Anti-TT IgG and anti-DT IgG and seroprotection rate at delivery in maternal participants and in infants at time of birth and at 2 months of age.

Maternal antibody concentration								
	**TT**				**DT**			
	**Tdap1_gen_**	**Tdap2_gen_**	**TdaP5_gen_**	**Tdap8_chem_**	**Tdap1_gen_**	**Tdap2_gen_**	**TdaP5_gen_**	**Tdap8_chem_**
**N**	77	79	75	78	77	79	75	78
**Baseline** GMC (IU/mL)	1.0	0.9	0.8	0.8	0.1	0.1	0.1	0.1
(95% CI)	(0.8**–**1.2)	(0.7**–**1.1)	(0.7**–**1.1)	(0.6–1.1)	(0.1–0.1)	(0.1–0.1)	(0.1–0.1)	(0.1–0.1)
**Delivery** GMC (IU/mL)	2.6	3.2	2.5	3.1	0.4	0.6	0.6	0.6
(95% CI)	(2.3**–**3.0)	(2.7**–**3.8)	(2.2**–**2.9)	(2.6–3.7)	(0.3–0.5)	(0.4–0.7)	(0.4–0.8)	(0.5–0.8)
**Delivery/baseline** GMFR^1^	2.6	3.7	3.0	3.8	4.0	7.5	6.2	6.9
(95% CI)	(2.1**–**3.3)	(2.7**–**5.0)	(2.2**–**4.1)	(2.7–5.2)	(3.2–4.9)	(6.1–9.3)	(4.5–8.5)	(5.5–8.6)
**Infant antibody concentration**								
**Time of birth**^2^**, N**	76	79	76	78	76	79	76	78
GMC (IU/mL)	3.5	4.6	3.7	4.6	0.5	0.8	0.7	0.8
(95% CI)	(3.0**–**4.0)	(3.9**–**5.4)	(3.2**–**4.2)	(3.8–5.4)	(0.4–0.6)	(0.6–1.0)	(0.5–1.0)	(0.6–1.0)
**Time of birth/delivery**								
**GMC or GMT ratio**^3^	1.3	1.5	1.5	1.5	1.2	1.3	1.3	1.3
(95% CI)	(1.2**–**1.4)	(1.4**–**1.5)	(1.4**–**1.5)	(1.4–1.6)	(1.1–1.3)	(1.1–1.6)	(1.0–1.5)	(1.2–1.4)
**2 months, N**	73	76	73	74	73	76	73	74
GMC (IU/mL)	1.0	1.2	1.0	1.3	0.1	0.2	0.2	0.2
(95% CI)	(0.9**–**1.2)	(1.0**–**1.4)	(0.9**–**1.2)	(1.0–1.5)	(0.1–0.2)	(0.1–0.2)	(0.1–0.2)	(0.2–0.2)
**Maternal seroprotection rate (anti-TT IgG and anti-DT IgG ≥ 0.1 IU/mL)**								
**Delivery, N**	77	79	75	78	77	79	75	78
n (%)	77 (100.0)	79 (100.0)	75 (100.0)	78 (100.0)	71 (92.2)	73 (92.4)	68 (90.7)	75 (96.2)
(95% CI)	(95.3**–**100.0)	(95.4**–**100.0)	(95.2**–**100.0)	(95.4–100.0)	(83.8–97.1)	(84.2–97.2)	(81.7–96.2)	(89.2–99.2)
**Infant seroprotection rate (anti-TT IgG and anti-DT IgG ≥ 0.1 IU/mL)**								
**Time of birth**^2^**, N**	76	79	76	78	76	79	76	78
n (%)	76 (100.0)	79 (100.0)	76 (100.0)	78 (100.0)	70 (92.1)	76 (96.2)	73 (96.1)	74 (94.9)
(95% CI)	(95.3**–**100.0)	(95.4**–**100.0)	(95.3**–**100.0)	(95.4**–**100.0)	(83.6–97.1)	(89.3–99.2)	(88.9–99.2)	(87.4–98.6)
**2 months, N**	73	76	73	74	73	76	73	74
n (%)	73 (100.0)	76 (100.0)	73 (100.0)	74 (100.0)	49 (67.1)	57 (75.0)	52 (71.2)	58 (78.4)
(95% CI)	(95.1**–**100.0)	(95.3**–**100.0)	(95.1**–**100.0)	(95.1**–**100.0)	(55.1–77.7)	(63.7–84.2)	(59.5–81.2)	(67.3–87.1)

Abbreviations: CI, confidence interval; DT, diphtheria toxoid; GMC, geometric mean concentration; GMFD, geometric mean fold difference; GMFR, geometric mean fold rise from baseline; IgG, immunoglobulin G; IU, international unit; mL, milliliter; N or n, number of participants; TT, tetanus toxoid.

1 Geometric mean fold rise (GMFR) is geometric mean of the ratios of antibody concentration at delivery to antibody concentration at baseline.

2 Time of birth (cord blood or neonatal blood within 72 hours after birth).

3 The ratio of study vaccine group (Tdap1_gen_/Tdap2_gen_/TdaP5_gen_) to comparator group (Tdap8_chem_).

In infants at birth, anti-PT GMCs ranged from 37.2 (95% CI 30.6–45.2) in Tdap1_gen_ group to 118.8 (95% CI 93.9–150.4 in the TdaP5_gen_ group. These levels were consistently higher in cord blood or neonatal samples compared to maternal blood, demonstrating active transport of antibodies from maternal participants to their infant. Interestingly, the anti-PT GMCs in all groups were ≥30 IU/mL, a cut-off value considered potentially predictive of protection of infants until 2 to 3 months of age based on a half life of 36 days [23].

In infants at 2 months of age, anti-PT GMCs (IU/mL) ranged from 10.5 (95% CI 8.6–12.9) in the Tdap1_gen_ group to 32.8 (95% CI 25.7–42.0) in the TdaP5_gen_ group. This highlights significantly higher anti-PT IgG concentration in TdaP5_gen_ than Tdap8_chem_ (12.5 [95% CI 10.1–15.5], p<0.05). For anti-FHA, the GMC values ranged from 16.6 (95% CI 13.8–19.8) in the Tdap1_gen_ group to 54.8 (95% CI 43.2–69.3) in Tdap8_chem._ For PTNA, there was no significant difference at 2 months between the recombinant vaccines and the comparator with the following GMTs: 11.8 (95% CI 6.4–21.5) in the ap1_gen_ group, 12.5 (95% CI (8.0–19.6) in the Tdap8_chem_ group and 28.7 (95% CI 18.1–45.6) in the TdaP5_gen_ group (Table 4).

In infants at 2 months of age, anti-TT seroprotection rates were 100% across all groups and anti-DT rates were highest for Tdap8_chem_ group (78.4% [95% CI 67.3–87.1]) and lowest for Tdap1_gen_ (67.1% [95% CI 55.1–77.7]) (Table 5). For pertussis, all infant groups had anti-PT GMC ≥30 IU/mL at birth and ≥10 IU/mL at 2 months of age. Even though there is no well-defined anti-PT concentration that correlates with protection, the birth cut-off value of ≥30 IU/mL was considered potentially predictive of protection of infants until 2 to 3 months of age based on a half life of 36 days [23].

**Fig. 2:**
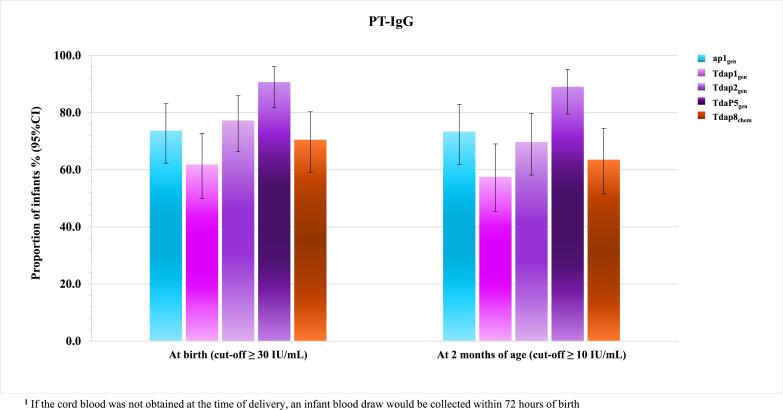
Proportion of infants with cut-off levels of anti-PT antibody at birth^1^ and at 2 months of age.

The proportion of infants at birth with cut-off value of ≥30 IU/mL for anti-PT antibody concentration ranged from 61.8% (Tdap1_gen_ group) to 90.7% (Boostagen®) (Fig. 2). At 2 months of age, the proportion of infants with cut-off value of ≥10 IU/mL for anti-PT antibody concentration ranged from 57.3% (Tdap1_gen_ group) to 89.0% (Boostagen®) (Fig. 2).

A comparison of GMCs of anti-PT antibodies and GMTs of PT neutralizing antibody at the time of birth (cord blood or neonatal blood ≤72 hours after birth) in maternal participants vaccinated during the second versus third trimester of pregnancy. At second trimester of pregnancy, the anti-PT antibodies ranged from 33.03 IU/mL (95% CI 24.05–45.38) for Tdap1_gen_ to 113.36 IU/mL (95% CI 82.82–155.17) for TdaP5_gen_, and at third trimester, the range are from 41.17 IU/mL (95% CI 32.15–52.73) for Tdap1_gen_ to 123.66 IU/mL (95% CI 86.56–176.67) for TdaP5_gen_ vaccine group. Similar values were obtained for PT neutralizing antibody. No difference in these outcomes between the second and third trimester of pregnancy was observed.

### 3.3 Safety of maternal participants and infants

An overview of safety results in maternal participants until 2 months after delivery is presented in Table 6. There were no vaccine-related SAEs, no AE leading to study withdrawal, and no deaths. Between 21.5% and 38.0% of maternal participants in each study arm experienced at least one complication during pregnancy. Furthermore, between 21.3% and 35.4% of participants in each vaccine group had at least one complication during labor or delivery. Please refer to Table 7 for data regarding specific diagnoses. The percentage of maternal participants who had caesarean section ranged from 41.3% (TdaP5_gen_ group) to 53.8% (Tdap1_gen_ group), with a history of previous caesarean section as the predominant reason leading to caesarean section in the study. In infants, the outcome at birth is shown in Table 6 with 9.6% (38/398) presenting with one or more of either prematurity, SGA, or low birthweight, and ranging from 1.3% (1/79) for Tdap8_chem_ to 16.3% (13/80) for Tdap1_gen_. Overall, prematurity was the most frequently reported outcome at 7.5% (30/398). Three cases of congenital anomalies were reported (0.8%) in this study. One or more non vaccine-related SAEs were reported in 13.7% (54/393) of eligible infants. No SAEs led to death or study withdrawal.

**Table 6:** Medically attended adverse events and serious adverse events in maternal participants during pregnancy (from baseline, delivery and until 2 months after delivery), and outcome in infants at birth.

**Medically attended adverse events and serious adverse events in maternal participants (baseline until 2 months after delivery)**	**ap1_gen_** **n (%), E**	**Tdap1_gen_** **n (%), E**	**Tdap2_gen_** **n (%), E**	**TdaP5_gen_** **n (%), E**	**Tdap8_chem_** **n (%), E**	**Total** **n (%), E**
Number of maternal participants	80	80	80	80	80	400
With medically attended adverse event	11 (13.8), 14	18 (22.5), 28	16 (20.0), 19	15 (18.8), 19	13 (16.3), 17	73 (18.3), 97
With one or more serious adverse events	8 (10.0), 9	17 (21.3), 29	11 (13.8), 14	13 (16.3), 17	7 (8.8), 9	56 (14.0), 78
With vaccine-related serious adverse events	0 (0.0), 0	0 (0.0), 0	0 (0.0), 0	0 (0.0), 0	0 (0.0), 0	0 (0.0), 0
Death	0 (0.0), 0	0 (0.0), 0	0 (0.0), 0	0 (0.0), 0	0 (0.0), 0	0 (0.0), 0
Life-threatening	0 (0.0), 0	0 (0.0), 0	0 (0.0), 0	0 (0.0), 0	1 (1.3), 3	1 (0.3), 3
Inpatient hospitalization/prolongation of existing hospitalization	6 (7.5), 6	12 (15.0), 23	9 (11.3), 10	9 (11.3), 11	5 (6.3), 5	41 (10.3), 55
Persistent or significant disability or incapacity	0 (0.0), 0	0 (0.0), 0	0 (0.0), 0	0 (0.0), 0	0 (0.0), 0	0 (0.0), 0
Congenital anomaly or birth defect in the offspring of subject	0 (0.0), 0	0 (0.0), 0	0 (0.0), 0	0 (0.0), 0	0 (0.0), 0	0 (0.0), 0
Medically significant adverse event	4 (5.0), 4	9 (11.3), 13	5 (6.3), 6	8 (10.0), 12	2 (2.5), 2	28 (7.0), 37
**Pregnancy, delivery and infant outcomes**	**ap1_gen_** **n (%)**	**Tdap1_gen_** **n (%)**	**Tdap2_gen_** **n (%)**	**TdaP5_gen_** **n (%)**	**Tdap8_chem_** **n (%)**	**Total** **n (%)**
**Pregnancy and delivery outcome, N**	**79**^1^	**80**	**79**	**80**	**79**	**397**
Delivery mode						
Vaginal	39 (49.4)	37 (46.3)	43 (54.4)	47 (58.8)	38 (48.1)	204 (51.4)
Caesarean section	40 (50.6)	43 (53.8)	36 (45.6)	33 (41.3)	41 (51.9)	193 (48.6)
Complication during pregnancy	24 (30.4)	26 (32.5)	30 (38.0)	24 (30.0)	17 (21.5)	121 (30.5)
Complication during labor or delivery	26 (32.9)	27 (33.8)	28 (35.4)	17 (21.3)	22 (27.9)	120 (30.2)
**Infant outcome, N**	**80**	**80**	**79**	**80**	**79**	**398**
Infants with ≥1 criteria for exclusion	5 (6.3)	13 (16.3)	9 (11.4)	10 (12.5)	1 (1.3)	38 (9.6)
Prematurity^2^	4 (5.0)	10 (12.5)	8 (10.1)	8 (10.0)	0 (0.0)	30 (7.5)
Small for gestational age	2 (2.5)	4 (5.0)	1 (1.3)	3 (3.8)	1 (1.3)	11 (2.8)
Low birth weight^3^	1 (1.3)	3 (3.8)	0 (0.0)	1 (1.3)	0 (0.0)	5 (1.3)
**Serious adverse events in infants (at birth)**	**ap1_gen_** **n (%), E**	**Tdap1_gen_** **n (%), E**	**Tdap2_gen_** **n (%), E**	**TdaP5_gen_** **n (%), E**	**Tdap8_chem_** **n (%), E**	**Total** **n (%), E**
Eligible infants, N	79	77	79	79	79	393
Eligible infants with ≥1 serious adverse event	10 (12.7), 13	14 (18.2), 25	9 (11.4), 11	14 (17.7), 21	7 (8.9), 9	54 (13.7), 79
Death	0 (0.0), 0	0 (0.0), 0	0 (0.0), 0	0 (0.0), 0	0 (0.0), 0	0 (0.0), 0
Life-threatening	1 (1.3), 1	0 (0.0), 0	0 (0.0), 0	0 (0.0), 0	0 (0.0), 0	1 (0.3), 1
Inpatient hospitalization / prolongation of existing hospitalization	6 (7.6), 7	9 (11.7), 14	3 (3.8), 3	3 (3.8), 6	5 (6.3), 7	26 (6.6), 37
Persistent or significant disability or incapacity	0 (0.0), 0	0 (0.0), 0	0 (0.0), 0	0 (0.0), 0	0 (0.0), 0	0 (0.0), 0
Congenital anomaly or birth defect in the offspring of subject	0 (0.0), 0	1 (1.3), 3	1 (1.3), 1	1 (1.3), 2	0 (0.0), 0	3 (0.8), 6
Medically significant adverse event	6 (7.6), 8	8 (10.4), 16	7 (8.9), 7	12 (15.2), 18	4 (5.7), 5	37 (9.4), 54
Neonatal blood screening abnormalities	0 (0.0), 0	0 (0.0), 0	0 (0.0), 0	0 (0.0), 0	0 (0.0), 0	0 (0.0), 0
Hearing deficiency detected through neonatal screening	0 (0.0), 0	0 (0.0), 0	0 (0.0), 0	0 (0.0), 0	0 (0.0), 0	0 (0.0), 0

Abbreviations: E, number of events; g, gram; N or n, number of participants with the event.

1 Missing information in a maternal participant who delivered in another hospital.

2 < 37 weeks gestational age

3 < 2000 g

**Table 7:** Summary of pregnancy complications.

**Pregnancy complication**	**ap1_gen_** **(N=79)**	**Tdap1_gen_** **(N=80)**	**Tdap2_gen_** **(N=79)**	**TdaP5_gen_** **(N=80)**	**Tdap8_chem_** **(N=79)**
	**n (%)**	**n (%)**	**n (%)**	**n (%)**	**n (%)**
Pregnancy loss or stillbirth	0	0	0	0	0
Preterm delivery	4 (5.0)	10 (12.5)	8 (10.1)	8 (10.0)	0
Premature rupture of membranes	3 (3.8)	7 (8.8)	7 (8.9)	5 (6.3)	3 (3.8)
Pregnancy-induced hypertension	1 (1.3)	4 (5.0)	5 (6.3)	1 (1.3)	3 (3.8)
Preeclampsia/eclampsia	1 (1.3)	4 (5.0)	1 (1.3)	2 (2.5)	2 (2.5)
Intrauterine growth restriction	1 (1.3)	0	0	1 (1.3)	0
Obstetric hemorrhage	1 (1.3)	0	0	0	0
Gestational diabetes	8 (10.0)	8 (10.0)	9 (11.4)	6 (7.5)	3 (3.8)
Other	10 (12.5)	4 (5.0)	8 (10.1)	10 (12.5)	9 (11.4)
Acute nasopharyngitis	1 (1.3)	0	0	0	0
Anemia	0	0	1 (1.3)	0	0
Cellulitis	0	0	1 (1.3)	0	0
Fetal distress	5 (6.3)	2 (2.5)	2 (2.5)	3 (3.8)	7 (8.9)
Fever	1 (1.3)	0	0	0	0
Hematuria	0	0	0	1 (1.3)	0
Infection	2 (2.5)	0	1 (1.3)	2 (2.5)	0
Myoma	0	0	1 (1.3)	1 (1.3)	0
Oligohydramnios	1 (1.3)	1 (1.3)	0	2 (2.5)	1 (1.3)
Placenta previa	0	1 (1.3)	0	0	0
Threatened preterm labor	0	0	2 (2.5)	1 (1.3)	1 (1.3)

Abbreviation: N or n, number of participants.

## 4 Discussion

We evaluated the pregnancy outcome and antibody transfer to neonates after maternal immunization with recombinant pertussis vaccines containing different concentrations of genetically inactivated pertussis toxin. We found that anti-PT antibody levels at delivery were similar or higher across multiple dose levels compared to a reference vaccine known to protect against neonatal pertussis. Anti-PT, anti-FHA, anti-DT, and anti-TT GMC slightly decreased from 28 days after vaccine administration to the time of delivery with a single dose of recombinant acellular pertussis vaccine (either ap1_gen_, Tdap1_gen_, Tdap2_gen_, or TdaP5_gen_) in the second or third trimester of pregnancy. The GMCs were higher through the time of delivery than the GMC levels reported before vaccine administration. Immunogenicity data in infants reflected the pattern of results observed in the maternal participants; antibody titers were slightly higher than in maternal participants, indicating an active antibody transfer from the mother to the infant, and persistent with effective antibody levels up to 2 months of age.

No safety issues of concern were identified in the study. Evaluation of pregnancy and neonatal outcomes in mothers and infants showed no vaccine-related adverse effects on pregnancy or newborn health outcomes. Adverse pregnancy and neonatal outcomes in infants were similar across all vaccine groups. In our study, the incidence rate of prematurity or preterm birth (7.5%) and the rate of caesarean section (48.6%) were found similar to that in the general population at the two sites. There was no difference in proportion of caesarian section between vaccine groups. The high rate of caesarean sections in Thailand can be due to fear of labor pain [24] and belief in “auspicious dates” [25].

The ratio of anti-PT antibody from cord blood to delivery, in which GMCs were consistently higher in cord blood or neonatal samples than in maternal blood, demonstrates the active transfer of antibodies from mothers to their infants across all recombinant pertussis vaccine formulations [26], [27], [28].

Of note, pertussis vaccination is recommended in the third trimester of gestation by the American College of Obstetricians and Gynecologists [6] while it is recommended in the second or third trimester in other countries [8], [29]. Previous studies have investigated the optimal timing of maternal Tdap vaccination indicating that administration in the second or third trimester results in relatively higher neonatal antibody concentrations [23], [30], [31]. The evidence to date has been inconclusive regarding which trimester is preferable. However, in one prospective, observational, nonrandomized study comparing the transfer of anti-PT antibodies to the newborn following vaccination in the second and third trimester of pregnancy, second trimester vaccination conferred higher antibody concentrations to the newborn, particularly for premature babies [31].

In our study, we found no difference in GMC of anti-PT antibodies and GMT of PTNA at the time of birth if the vaccine was given during the second versus third trimester of pregnancy. These findings provide useful information regarding programmatic suitability for maternal pertussis immunization implementation, as the studied vaccines can be given at any time during the second or third trimester of gestation and at least fifteen days before delivery, as per World Health Organisation recommendations.

When comparing Tdap1_gen_ and Tdap2_gen_ to TdaP5_gen_, we have observed a dose-dependent immune response against PT antigen in mothers and infants. Among all vaccine groups including Tdap8_chem_, the immune response to FHA was also dose dependent with titers increasing according to FHA content (1 µg, 5 µg or 8 µg). When compared to Tdap8_chem_, Tdap1_gen_ induces the same anti-PT GMC with an adjusted GMC ratio of 1 (95% CI, 0.7–1.4), demonstrating PT_gen_ is a strong immunogen even at a very low concentration (1 µg PT_gen_) [12], [11], [14], [19].

In addition, vaccination with monovalent ap1_gen_ induced higher anti-PT GMC than Tdap1_gen_ in mothers and infants up to 2 months of age. This difference was found significant only when using adjusted GMC in mothers at delivery. This may be explained by potential interference of diphtheria and tetanus toxoids on anti-pertussis responses. Interestingly, when compared to Tdap8_chem_, the ap1_gen_ vaccine induced significantly higher PT levels at the time of delivery, confirming the non-inferiority and superiority of ap1_gen_ demonstrated in non-pregnant and pregnant women, one month after vaccination [19]. Given these findings, monovalent acellular pertussis vaccine could potentially be used for subsequent pregnancies that do not need the diphtheria and tetanus components, avoiding unnecessary reactogenicity and cost of Td-combined formulations.

We also found that in 2 month-old infants, levels of PT IgG elicited by recombinant pertussis formulations were higher than non-recombinant Tdap comparator, Tdap8 _chem_, which has been shown to be effective in reducing neonatal pertussis in observational studies [5].

Higher titers against (PT) elicited by the recombinant vaccines would be expected to increase protection against severe pertussis in young infants. A Consensus Conference organized by the World Association for Infectious Disease and Immunological Disorders (WAidid), with the goal of evaluating the most important reasons for the pertussis resurgence and the role of different acellular pertussis vaccines in this resurgence concluded that present knowledge indicates that PT, particularly if genetically detoxified, represents the main antigen that ensures protection from disease, although not from infection; and that the contribution of other pertussis antigens (FHA, PRN and FIM) in vaccine efficacy and long-lasting protection is still under discussion and needs further study [32].

PT_gen_ containing vaccines (aP5_gen_, TdaP5_gen_ and Tdap2_gen_) are licensed for booster use in adolescents and adults including pregnant women and the elderly. The availability of affordable low-dose ap1_gen_ and Tdap1_gen_ vaccines could make maternal pertussis vaccination more accessible in low-middle income countries.

## Funding

This work was funded by a grant from the Bill & Melinda Gates Foundation, Seattle, USA [grant number OPP1120084]. The findings and conclusions contained within are those of authors and do not reflect position or policies of the Bill & Melinda Gates Foundation.

## CRediT authorship contribution statement

**Kulkanya Chokephaibulkit:** Supervision, writing – review & editing. **Thanyawee Puthanakit:** Supervision, writing – review & editing. **Surasith Chaithongwongwatthana:** Investigation, writing – review & editing. **Niranjan Bhat:** Conceptualization, methodology, writing – review & editing. **Yuxiao Tang:** Conceptualization, methodology, writing – review & editing. **Suvaporn Anugulruengkitt:** Investigation, writing – review & editing. **Chenchit Chayachinda:** Investigation, writing – review & editing. **Sanitra Anuwutnavin:** Investigation, writing – review & editing. **Keswadee Lapphra:** Investigation, writing – review & editing. **Supattra Rungmaitree:** . **Monta Tawan:** Investigation. **Indah Andi-Lolo:** Writing – review & editing. **Renee Holt:** Writing – review & editing. **Librada Fortuna:** Methodology, investigation, validation, writing – review & editing. **Chawanee Kerdsomboon:** Investigation, validation, writing – review & editing, visualization. **Vilasinee Yuwaree:** Investigation, writing – review & editing. **Souad Mansouri:** Conceptualization, methodology, supervision, writing – review & editing. **Pham Hong Thai:** Conceptualization, methodology, resources, writing – review & editing. **Bruce L. Innis:** Conceptualization, methodology, writing – review & editing.

## Declaration of competing interest

The authors declare the following financial interests/personal relationships which may be considered as potential competing interests: LF, CK, VY, SM and PHT are employed by BioNet. All other authors declare no competing interests.

## Data Availability

Data will be made available on request.
