# Supplementary material for: Effective and safe transfer of maternal antibodies persisting two months postpartum following maternal immunization with different doses of recombinant pertussis-containing vaccines
Source: Vaccine. 2024 Jan 12;42(2):383–95. doi: 10.1016/j.vaccine.2023.11.042 (PMC10789266; doi:10.1016/j.vaccine.2023.11.042)
Supplement: Supplementary data 1 [file mmc1.docx]

**Supplementary Appendix**

#### Exclusion criteria for pregnant women

The exclusion criteria included any significant congenital abnormality as documented by ultrasound or a history of significant medical illness such as immune deficiency, hypertension or other cardiovascular disease, or renal or hepatic diseases. Women with pregnancy complications in the current pregnancy, such as hypertension (blood pressure >140/90 mmHg in the presence of proteinuria or blood pressure >150/100 mmHg with or without proteinuria) or currently on an antihypertensive therapy, or preeclampsia. Those with endocrine disorders, including (but not limited to) hyperthyroidism, untreated hypothyroidism, and glucose intolerance (e.g., diabetes mellitus type 1 or 2) antedating pregnancy, or occurring during pregnancy and requiring interventions other than diet for control were also excluded.

Other exclusion criteria included subjects with:

- Prior stillbirth or neonatal death, or multiple (≥3) spontaneous abortions.
- Prior preterm delivery <37 weeks gestation, or ongoing intervention (medical or surgical) in the current pregnancy to prevent preterm birth.
- Greater than five prior deliveries.
- History of allergy to any vaccine component.
- History of neurological adverse event after injection with any vaccine.
- Having received any other vaccines within 28 days prior to recruitment (3 months for live attenuated vaccines).
- History of receiving blood or blood component or immunoglobulin (except for prophylactic anti-Rho (D) immune globulin) within 6 months prior to recruitment.
- History of receiving immunosuppressive drugs or systemic corticosteroid (>0.5 milligram (mg)/kilogram (kg) of prednisolone or equivalent for more than 14 days) within 3 months prior to recruitment.
- Having received diphtheria, or tetanus, or pertussis-containing vaccine, or experienced a physician-diagnosed pertussis illness within 1 year prior to recruitment.
- Any progressive or severe neurological disorder, seizure disorder, or with history of Guillain-Barré syndrome.
- Behavioral or cognitive impairment, or psychiatric disease that, in the opinion of the investigator, could have interfered with the subject's ability to participate in the study.
- History of smoking, alcoholism, and/or intravenous drug abuse which in the opinion of the investigator could have interfered with the study outcome evaluation.
- Presence of bleeding disorders, including deep venous thrombosis or thromboembolism, or the use of anticoagulants during pregnancy, and abnormalities of splenic and thymic functions.
- With history of any illness or condition that, in the opinion of the investigator or obstetrician, would classify the pregnancy as high-risk, interfere with the results of the study, or pose additional risk to the mother or fetus/infant due to participation in the study.

**Exclusion criteria for infants**

If an infant meets ANY of the following criteria at birth, vaccination may be delayed or suspended according to investigator judgement. In this case, the infant will be referred to his/her primary care provider and the mother-infant pair will continue to be monitored for safety up to Visit 10 (the study end).

1. Birth weight ≤ 2,000 grams

2. Serious underlying medical condition*

3. Anything in the opinion of the investigator that would prevent the infant from participating in the study or would put the child at risk

*Serious underlying medical condition means any condition considered by the investigator to affect the infant’s participation in this study such as neonatal sepsis, infant respiratory distress syndrome, congenital heart disease, birth defect, non-physiologic jaundice, neonatal autoimmune thrombocytopenia, neurologic condition (such as neonatal convulsions), necrotizing enterocolitis, an intracranial hemorrhage and others.

**Ineligible infants excluded from safety analysis**

Five infants were excluded because of low birth weight (exclusion criteria no. 1). Of these 5 infants, 2 of them also met exclusion criteria no. 2.

**Maternal and infant participants excluded from immunogenicity analysis**

| **Reasons** | **ap1_gen_**  **n (%)** | **Tdap1_gen_**  **n (%)** | **Tdap2_gen_**  **n (%)** | **TdaP5_gen_**  **n (%)** | **TdaP8_chem_**  **n (%)** | **Total**  **n (%)** |
| --- | --- | --- | --- | --- | --- | --- |
| **Maternal participants excluded from immunogenicity analysis at delivery** |  |  |  |  |  |  |
| Participant received another vaccine before delivery | 1 (1.3)* | 1 (1.3) | 0 (0.0) | 1 (1.3) | 1 (1.3) | 4 (1.0) |
| Participant received another vaccine within 28 days prior to receiving the study vaccine | 1 (1.3) | 0 (0.0) | 0 (0.0) | 0 (0.0) | 0 (0.0) | 1 (0.3) |
| Participant received study vaccine at GA <20 weeks. | 0 (0.0) | 0 (0.0) | 0 (0.0) | 1 (1.3) | 0 (0.0) | 1 (0.3) |
| Participant blood sample was not collected | 1 (1.3) | 2 (2.5) ** | 0 (0.0) | 3 (3.8)** | 0 (0.0) | 6 (1.5) |
| Participant withdrew consent before delivery | 0 (0.0) | 0 (0.0) | 0 (0.0) | 0 (0.0) | 1 (1.3) | 1 (0.3) |
| Participant migrated/moved from the study area before delivery | 0 (0.0) | 0 (0.0) | 1 (1.3)* | 0 (0.0) | 0 (0.0) | 1 (0.3) |
| **Infant participants excluded from immunogenicity analysis at delivery** | | | | | | |
| Mother received another vaccine before delivery | 1 (1.3)* | 1 (1.3) | 0 (0.0) | 1 (1.3) | 1 (1.3) | 4 (1.0) |
| Mother received another vaccine within 28 days prior to receiving the study vaccine | 1 (1.3) | 0 (0.0) | 0 (0.0) | 0 (0.0) | 0 (0.0) | 1 (0.3) |
| Mother received study vaccine at GA <20 weeks | 0 (0.0) | 0 (0.0) | 0 (0.0) | 1 (1.3) | 0 (0.0) | 1 (0.3) |
| Cord blood/infant blood sample was not collected | 2(2.5) | 3 (3.8)** | 0 (0.0) | 2 (2.5) * | 0 (0.0) | 7 (1.8) |
| **Infant participants excluded from immunogenicity analysis at 2 months of age** | | | | | | |
| Mother received another vaccine before delivery | 1 (1.3)* | 1 (1.3) | 0 (0.0) | 1 (1.3) | 1 (1.3) | 4 (1.0) |
| Mother received another vaccine within 28 days prior to receiving the study vaccine | 1 (1.3) | 0 (0.0) | 0 (0.0) | 0 (0.0) | 0 (0.0) | 1 (0.3) |
| Mother received study vaccine at GA <20 weeks | 0 (0.0) | 0 (0.0) | 0 (0.0) | 1 (1.3) | 0 (0.0) | 1 (0.3) |
| Infant participant received DTaP vaccine | 1 (1.3) | 0 (0.0) | 0 (0.0) | 0 (0.0) | 0 (0.0) | 1 (0.3) |
| Infant participant received DTwP vaccine before blood collection | 0 (0.0) | 2 (2.6) | 1 (1.3) | 2 (2.5) | 0 (0.0) | 5 (1.3) |
| Infant participant received hepatitis B immunoglobulin (HBIG) at delivery | 0 (0.0) | 0 (0.0) | 0 (0.0) | 0 (0.0) | 1 (1.3) | 1 (0.3) |
| Infant participant blood sample was not collected | 1 (1.3) | 1 (1.3)* | 0 (0.0) | 0 (0.0) | 1 (1.3) | 3 (0.8) |
| Mother and infant migrated/moved from the study area after delivery | 0 (0.0) | 0 (0.0) | 2 (2.5) | 0 (0.0) | 1 (1.3) | 3 (0.8) |
| Mother and infant withdrew consent after delivery | 0 (0.0) | 0 (0.0) | 0 (0.0) | 1 (1.3) | 1 (1.3) | 2 (0.5) |
| Infant participant lost to follow-up after delivery | 0 (0.0) | 0 (0.0) | 0 (0.0) | 1 (1.3) | 0 (0.0) | 1 (0.3) |

* One participant was excluded from anti-PT, anti-FHA, anti-TT and anti-DT IgG antibodies (ELISA) and PT-neutralizing antibody titers (CHO cell assay).

** Two participants were excluded from anti-PT, anti-FHA, anti-TT and anti-DT IgG antibodies (ELISA) and PT-neutralizing antibody titers (CHO cell assay).

#### Immunogenicity outcomes

The immunogenicity outcomes for maternal and infant subject study groups (ap1_gen_, Tdap1_gen_, Tdap2_gen_ or TdaP5_gen_) and for the comparator group (Tdap8_chem_) were:

1. Comparison of GMC between study groups and comparator groups of anti-PT, anti-FHA, anti-DT and anti-TT antibodies and GMT of PT neutralizing antibody at delivery of maternal participants, and at the time of birth (cord blood or neonatal blood within 72 hours after birth) and 2 months of age in infants.
2. Comparison of seroconversion rate between study groups and the comparator group of anti-PT, anti-FHA antibodies and PT neutralizing antibody of maternal participants at delivery.
3. Comparison of seroprotection rate between study groups and comparator group of anti-DT and anti-TT antibodies in maternal participants at delivery, and at the time of birth (cord blood or neonatal blood within 72 hours after birth) and 2 months of age in infants.
4. Comparison of GMC of anti-PT antibodies and GMT of PT neutralizing antibody at 28 days after vaccination, delivery and at the time of birth (cord blood or neonatal blood within 72 hours after birth) in maternal participants vaccinated during the second trimester versus the third trimester of pregnancy.
5. Evaluation of antibody transfer from mothers to infants based on the ratio of GMC/GMT in infant cord blood or neonatal blood to that in maternal participants at the time of delivery in terms of anti-PT, anti-FHA, anti-diphtheria, and anti-tetanus antibody concentrations and PT neutralizing antibody titer.

The seroconversion rate for anti-PT, anti-FHA and PT neutralizing antibodies was defined as the proportion of maternal participants who achieved antibody concentration/titer increases of 4-fold or more from baseline to delivery. The seroprotection rate for anti-DT and anti-TT antibodies was defined by the proportion of subjects who had antibody concentrations ≥ 0.1 IU/mL at delivery for maternal participants, and at the time of birth (cord blood or neonatal blood within 72 hours after birth) and 2 months of age for infants.

#### Safety outcomes

The safety outcomes for maternal and infant subject study groups (ap1_gen_, Tdap1_gen_, Tdap2_gen_ or TdaP5_gen_) and for the comparator group (Tdap8_chem_) were:

1. Percentage of maternal participants with post vaccination onset of specific complications of pregnancy and delivery.
2. Percentage of maternal participants with MAAEs or SAEs reported from the day of vaccination until 2 months of infant age.
3. Percentage of infants with SAEs (including congenital anomalies, neonatal blood screening abnormalities, hearing deficiency detected through neonatal screening, and any other SAEs) occurring through 2 months of age.
4. Percentages of infant participants with prematurity (<37 weeks of gestation), small for gestational age (SGA) (<10^th^ percentile for gestational age)^1,^ or low birthweight (≤2000 g)

^1^ Schlaudecker EP, Munoz FM, Bardaji A, Boghossian NS, Khalil A, Mousa H, et al for the Brighton Collaboration Small

for Gestational Age Working Group. Vaccine 2017;35:6518-28. https://doi.org/10.1016/j.vaccine.2017.01.040.

####

#### Pregnancy and delivery complications

Pregnancy complications included pregnancy loss or stillbirth, preterm delivery (<37 weeks of gestation), premature rupture of membranes, pregnancy-induced hypertension, preeclampsia/eclampsia, intrauterine growth restriction, obstetric hemorrhage, and gestational diabetes. Labor and delivery complications included emergency caesarean section for maternal or fetal indications, postpartum hemorrhage, and maternal fever or infection.

**Definition of Medically Attended Adverse Event**

Medically attended adverse events (MAAEs) are events leading to an otherwise unscheduled visit to or from medical personnel for any reason, including visits to an accident and emergency department.

Source: Fulton TR, Narayanan D, Bonhoeffer J, Ortiz JR, Lambach P, Omer SB. A systematic review of adverse events following immunization during pregnancy and the newborn period. Vaccine 2015; 33:6453-65. https://doi.org/10.1016/j.vaccine.2015.08.043.

**Definition of Serious Adverse Event**

A serious adverse event (SAE) or reaction is any untoward medical occurrence that, at any dose:

• Results in death

• Is life-threatening

• Requires hospitalization or prolongation of existing inpatient’s hospitalization

• Results in persistent or significant disability or incapacity

• Is a congenital anomaly or birth defect in the offspring of a study subject

• Medically significant

Source: ICH Harmonised tripartite guideline. Clinical Safety Data Management: Definitions and Standards for Expedited Reporting. E2A. Current Step 4 version, dated 27 October 1994. Available from: https://database.ich.org/sites/default/files/E2A_Guideline.pdf.
